# The utility of MAS5 expression summary and detection call algorithms

**DOI:** 10.1186/1471-2105-8-273

**Published:** 2007-07-30

**Authors:** Stuart D Pepper, Emma K Saunders, Laura E Edwards, Claire L Wilson, Crispin J Miller

**Affiliations:** 1 Cancer Research UK, Paterson Institute for Cancer Research, The University of Manchester, Christie Hospital Site, Wilmslow Road, Withington, Manchester, M20 4BX, UK; 2 Epistem Ltd, 48 Grafton Street, Manchester, M13 9XX, UK

## Abstract

**Background:**

Used alone, the MAS5.0 algorithm for generating expression summaries has been criticized for high False Positive rates resulting from exaggerated variance at low intensities.

**Results:**

Here we show, with replicated cell line data, that, when used alongside detection calls, MAS5 can be both selective and sensitive. A set of differentially expressed transcripts were identified that were found to be changing by MAS5, but unchanging by RMA and GCRMA. Subsequent analysis by real time PCR confirmed these changes. In addition, with the Latin square datasets often used to assess expression summary algorithms, filtered MAS5.0 was found to have performance approaching that of its peers.

**Conclusion:**

When used alongside detection calls, MAS5 is a sensitive and selective algorithm for identifying differentially expressed genes.

## Background

A significant challenge with Affymetrix expression data is to provide an algorithm that combines the signals from the multiple Perfect-Match (PM) and Mismatch (MM) probes that target each transcript into a single value that sensitively and accurately represents its concentration. MAS5.0 does this by calculating a robust average of the (logged) PM-MM values [1]; increased variation is observed at low signal strengths and is at least in part due to the extra noise generated by subtracting the MM values from their PM partners [2].

A number of alternatives (e.g. RMA [3]) have been proposed that ignore the MM values, and consequently do not suffer from this source of variation. RMA successfully reduces the variance of low abundance transcripts and has been shown, using controlled datasets in which known quantities of specific mRNAs have been added to a common reference pool, to better distinguish differentially expressed transcripts from those that are unchanging [2-4]. In these experiments, only a small number of spikes were added, with the consequence that the vast majority of transcripts do not vary in concentration across the arrays, and that the MM signal, which results from the combined action of many weak, partial cross-hybridizations, might be expected to remain approximately constant [5]. However, whilst MM probes may not have a significant effect in 'spike-in' datasets such as these, they may be more useful with real experimental data, in which many more transcripts are changing. In a recent study, sets of 100–200 mRNAs were spiked into a known background set containing 2,551 different RNA species [5]. The authors found the MM probes to be of significantly more utility, and that MM probe subtraction as performed by MAS5.0 was the best background correction method of the algorithms on test. See also [6] for further consideration of these data. A variant of RMA, GCRMA [7,8], uses intensity-summaries generated across sets of probes to estimate non specific binding. The rationale is that hybridization signal (and therefore probe intensity) should at least in part be determined by binding efficiency. Irrespective of the way different algorithms calculate background, the consequences of a non-varying signal should still apply; Choe et al. found that the MAS5.0 MM subtraction outperformed the summary-approach of GCRMA on their dataset [5].

### Detection calls

In addition to expression summaries, the Affymetrix software also generates a p-score that assesses the reliability of each expression level. This is produced by using a signed rank test to consider the significance of the difference between the PM and MM values for each probeset [9]. Informally, MAS 5.0 can be seen to return two values, the first, an estimate of transcript concentration, and the second, a measure of how much the software 'believes' the first. Of potential confusion is the fact that this value is referred to as the 'detection p-value', and is subsequently used to generate a 'detection call', which flags the transcript as 'Present', 'Marginal' or 'Absent' (P/M/A). In fact, the detection p-value is closer to a reliability score – and the terms 'Reliable', 'Marginal' and 'Unreliable' might be more appropriate. The original approach to data analysis, proposed by the manufacturer, was to use the MAS5.0 expression summary to provide an estimate of transcript concentration, alongside detection calls to filter out unreliable probesets. Despite the fact that the expression summary algorithm has been shown to perform poorly on the test datasets described above, many researchers have continued to use this combined strategy to process their data. Shippy et al., for example, [10,11], use detection calls to eliminate unreliable data in a comparison between different array platforms and algorithms.

In the light of these recent findings, it is timely to revisit the performance of MAS5.0, paying particular attention to the use of detection calls. This paper describes a real-time PCR study designed to consider the differences between RMA, GCRMA and MAS5.0, with and without detection filtering, before revisiting the spike-in datasets described above. RMA and GCRMA were chosen for the study since they are widely used within the community and their behaviour is generally well understood. Comparing MAS5 to RMA allows it to be placed in context alongside other algorithms.

## Results

### Differential expression in replicated cell lines

**Figure 1:**
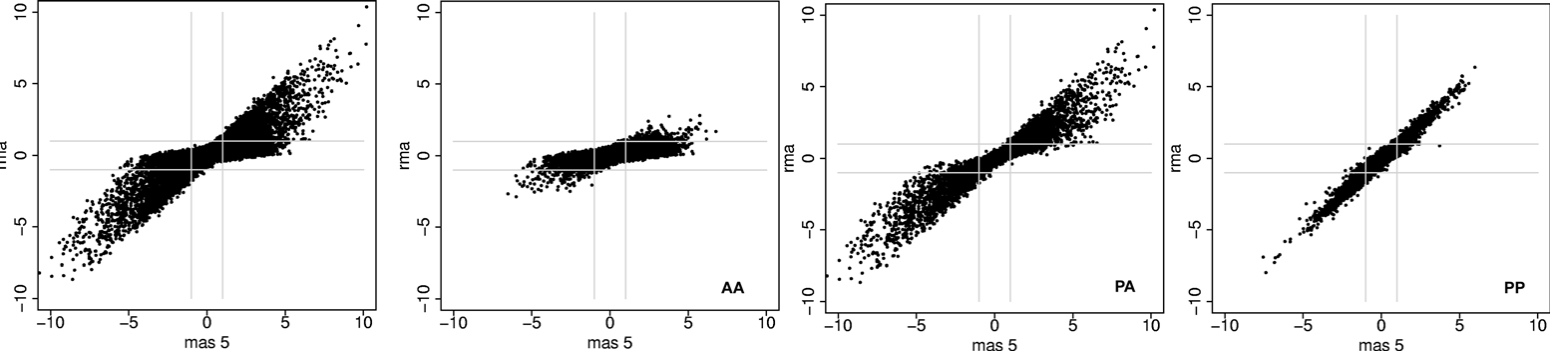
**A comparison of fold changes found by RMA and MAS5 for MCF7 and MCF10a cell line data**. Absent-flagged probesets show significant disagreement between the two algorithms. First panel: all data. Remaining panels are separated by detection calls. AA: probesets flagged Absent by MAS5 detection call. PA: probesets flagged Absent in one cell line, Present in the other. PP: probesets flagged Present in both cell lines. Lines represent 2-fold thresholds (data are on a log2 scale). Details of Present/Absent flagging can be found in Methods.

Figure 1 shows a comparison of the fold changes found using MAS5.0 and RMA between triplicate samples taken from two cell lines: the human breast cancer cell line MCF7, and the non-tumorigenic breast epithelial cell line, MCF10A, which unlike MCF7, lacks tumorigenicity in nude mice, three-dimensional growth in collagen, spontaneous- and anchorage-independent growth.

If both algorithms produced identical fold-changes, all points would be expected to lie on the diagonal. It can be seen that there is significant discrepancy between the fold changes reported by both algorithms, but that the majority of these variations are confined to probesets flagged Absent on one or more arrays. When data are stratified by detection call, it can be seen that for probesets flagged Present in all samples from both cell lines (PP), RMA and MAS5.0 report similar fold changes. For probesets that are flagged Absent on one or more arrays, there is disagreement (i.e. PA or AA in the figure), with RMA tending to report lower fold-changes than those found by MAS5.0. The graphs are also asymmetric. There are no probesets with high fold-changes according to RMA, but low fold-changes according to MAS5.0. GCRMA produced very similar results [additional file 1].

Figure 1 provides a qualitative overview of the data. Kappa coefficients [12] can provide an additional metric for the level of agreement between algorithms. Kappa is such that for complete agreement, *κ *= 1, for chance levels of agreement, *κ *= 0. With no stratification by detection call and a two-fold threshold, *κ *= 0.42 between algorithms. When analysis is restricted only to the PP probesets, agreement is significantly increased; *κ *= 0.82.

Figure 1 draws a distinction between probesets flagged Absent in both sets of samples (AA), and those flagged as Absent in one cell line, Present in the other (PA). If data are filtered using detection calls, then the AA probesets would be eliminated from further consideration. For these probesets, MAS5.0 and RMA are in agreement, except in cases where the fold change from RMA is greater than some significance threshold. Of the 29,998 (55%) AA probesets in this experiment, less than 2% (395) have an RMA fold change > 2. Thus RMA and MAS5.0 are in agreement for almost all AA probesets. The PA probesets in the figure represent those that are flagged as unreliable in one cell line, reliable in the other, and can be considered to be changing between being above and below the limit of detection for the system. For these, RMA consistently reports lower fold changes than MAS5.0 (1,522 of 6,296 probesets show at least a 2-fold lower fold change in RMA than MAS5.0). Over 18% of these probesets (1,142 of 6,296) have a fold change less than 2-fold by RMA but greater than 2-fold by MAS5.0.

Thus it can be seen that when data are stratified by detection call, only 1,522 + 395 = 1,917 probesets are called differently by MAS5 and RMA when a two fold cutoff is used for selection (*κ *= 0.80; RMA fold change > 2 vs. MAS5 fold change >2 and PP or PA). Although they represent a small proportion of the entire array, these probesets are of disproportionate interest because a substantial number of them are flagged as changing from Present to Absent by the detection calling algorithm, representing a change in concentration from above to below the levels of detection of the system. It is reasonable to consider the possibility that some of them, at least, are changing from 'on' to 'off'. This is a crucial subset of the data because of their potential to act as switches, invoking novel patterns of activity of phenotype.

### Validation by real time PCR

**Figure 2:**
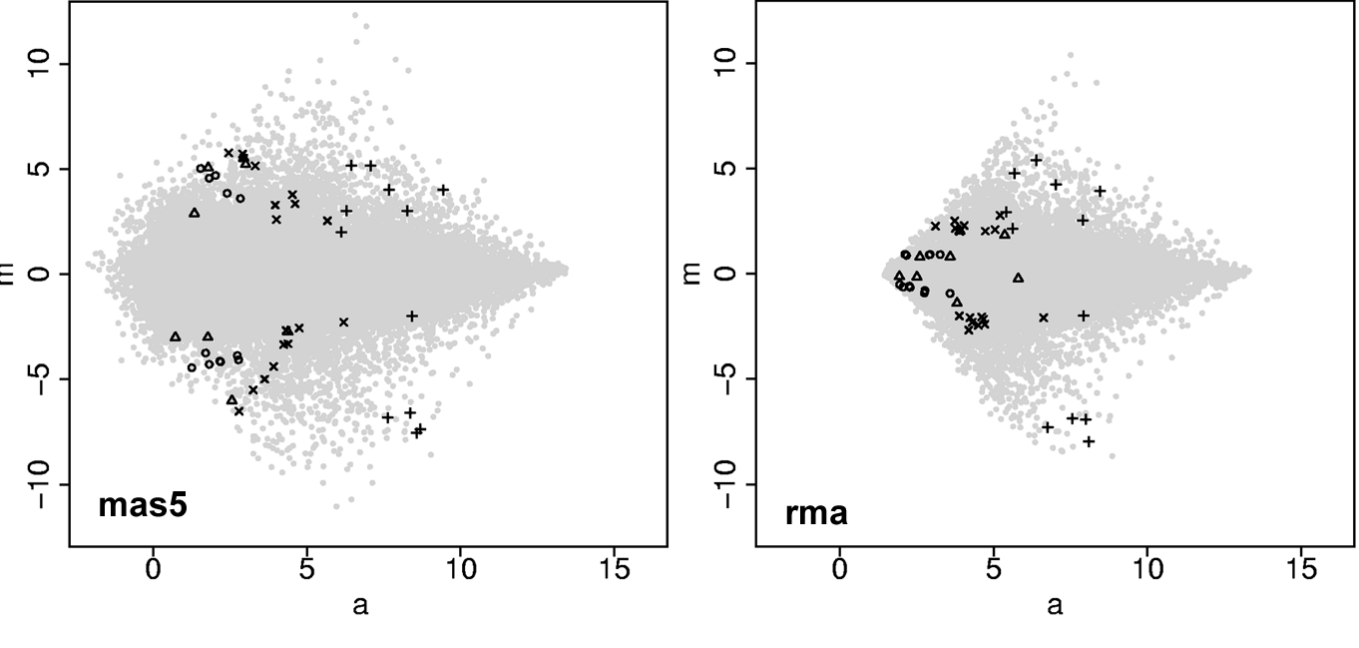
**MA plots of the MAS5 and RMA processed data showing probesets selected for real time PCR**. Light grey points all data. Circles: PA probesets. Diagonal Crosses: AA-RMA probesets. Triangles: AA probesets. Vertical Crosses: PP probesets. RMA reports the fold change for the PA, AA and AA-RMA probesets as low in comparison to MAS5. AA and PA probesets are of low intensity. Additional MA plots, stratified by detection call can be found in the supplementary data.

**Figure 3:**
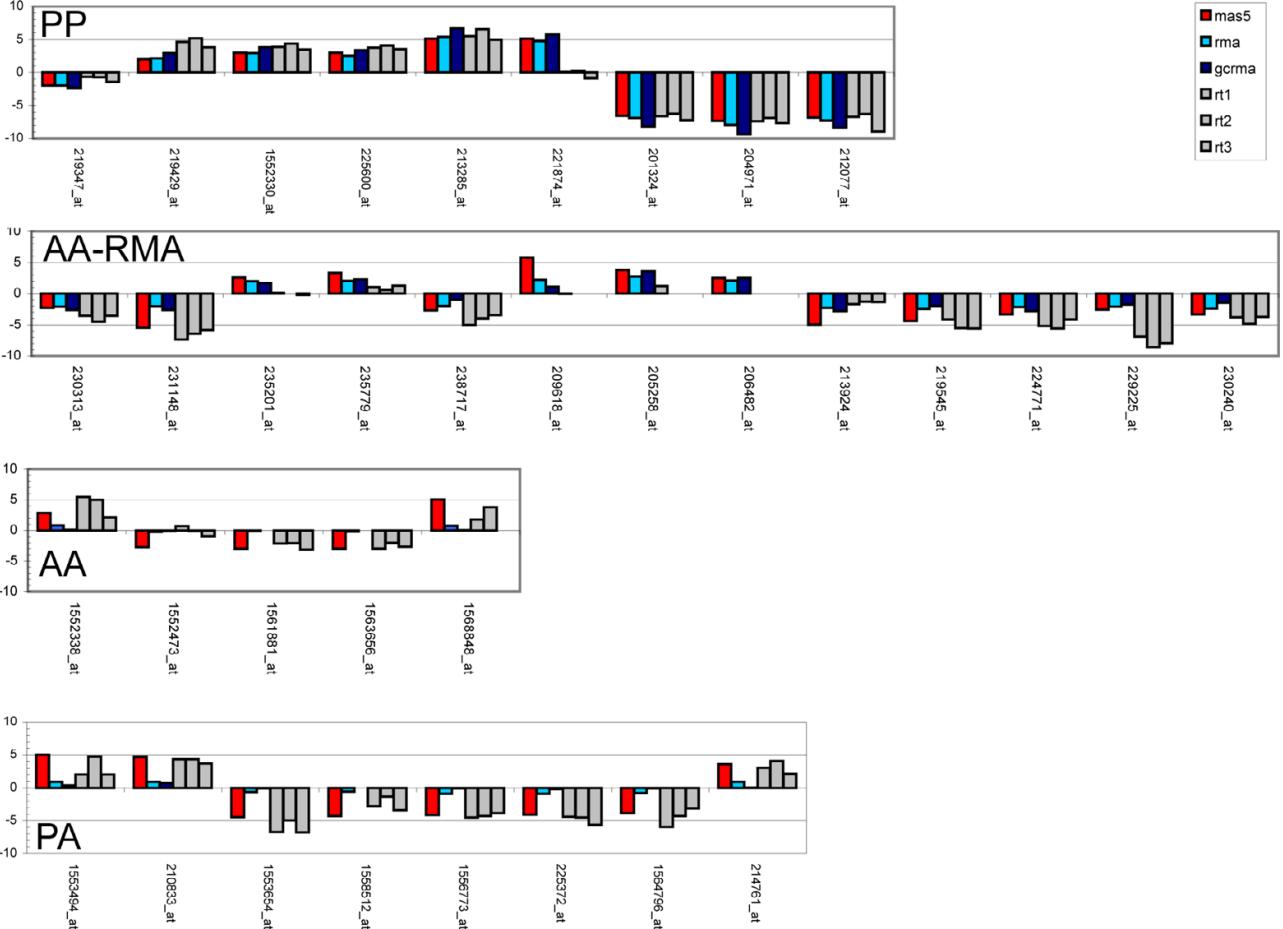
**Comparison between fold changes from MAS5 and RMA and those found using real time PCR (rt1, rt2, rt3)**. PA: Present-Absent probesets. PP: Present-Present probesets. AA: Absent-Absent probesets. AA-RMA: Absent-Absent probesets with fold changes > 2 according to RMA. Data are on a log2 scale.

In order to further investigate the differences between MAS5 and RMA, a set of probesets were selected for follow up by real time PCR, which was used to provide an independent estimate of the expected 'true' values [11]. Probesets were from taken from the PP, PA and AA sets, partitioned by fold change and selected to cover a range of fold changes (Methods; figure 2). Since the aim was to investigate the similarities and differences in fold change found by the different algorithms, data were stratified by fold change, not by intensity. Thus, four sets of probesets were considered: PP probesets that both RMA and MAS5 identified as changing, PA probesets (for which MAS5 reported a change and RMA reported small changes), AA probesets for which MAS5 reported low confidence in the data and RMA reported low fold change, and AA probesets for which RMA reported a fold-change greater than two (referred to here as 'AA-RMA' probesets). The 35 assays presented here represent PP, PA, AA and AA-RMA probesets across a range of fold changes. Figure 3 shows that for the PP probesets, real time PCR confirms the differential expression found using RMA, GCRMA and MAS5.0 for all but one probeset (221874_at). Even though the AA probesets were flagged as unreliable by detection call, they still receive a fold change from all three algorithms. All selected probesets had at least 4-fold differential expression according to MAS5.0 and a low fold change reported by RMA and GCRMA. In every case except one (1552473_at), real time PCR found significant fold changes in the same direction as that found by MAS5.0.

Thus, for the 5 AA probesets tested, all three algorithms correctly reported no change for one of the probes (1552473_at), and failed to correctly call fold changes for four of them (1552338_at, 1561881_at, 1563656_at, and 1568848_at). 395 probesets were found to have a >2 fold change by RMA but were flagged AA by MAS5. (Interestingly, 387 of them also had a fold change >2 reported by MAS5). 13 of these were tested by real time PCR, which found significant fold changes (i.e. greater than 2-fold), over all three replicates, for 9 of them and low fold changes for 1 (235201_at). For three assays (209618_at, 205258_at, 206482_at), real time PCR was unable to detect any expression. Thus, real time PCR supported the RMA and GCRMA data for 69% of the AA-RMA probesets.

Of the 1,522 PA probesets found to have less than 2-fold change according to RMA, 8 were pursued further by real time. For these, where GCRMA and RMA consistently reports lower fold changes than MAS5.0, the MAS5.0 data were supported by the real time PCR results in all 8 cases, contradicting the changes reported by the other algorithms. This is interesting not least because the detection calls are identifying these probesets as having poor signal to noise ratio for the Absent flagged data (see discussion).

It is useful to further quantify these results. One common method is to use Pearson correlation to provide a similarity metric. However, Pearson correlation (both centred and uncentred) must be treated with caution when comparing fold-changes. In particular, because it standardizes expression profiles, it is not appropriate if the actual magnitude of the fold-change is important, rather than simply the pattern of change across a set of samples. This is clearly demonstrated by the uncentred Pearson correlations for the MAS5 and RMA data, against the real time PCR results. For the PA probesets in figure 2, r = 0.93 for MAS5, and 0.92 for RMA, even though RMA consistently reports much lower fold-changes (max(fc): RMA = 0.92; MAS5 = 5.02; log_2 _scale). Correlation data for all subgroups can be found in [see additional file 2].

An alternate metric is the concordance correlation coefficient [13], r_c_. It is similar to Pearson's correlation, but measures not only the linear correlation between data points, but also how well they match the identity line [14]. Using r_c_, correlation is high for the PP probesets for both algorithms: r_c _= 0.92 and 0.93 for MAS5 and RMA, respectively. However, for the PA probesets, r_c _= 0.93 for MAS5, and 0.32 for RMA, for the AA-RMA probesets r_c _= 0.67 for MAS5 and 0.47 for RMA, and for the AA probesets, r_c _= 0.72 and 0.24, respectively. Thus the concordance correlation coefficient successfully distinguishes between the cases and provides a useful quantitative metric when fold changes are of importance.

It is also useful to consider these data in terms of True- and False- Positives and Negatives. In order to do this, a decision must be taken as to how to treat AA flagged data. For the purposes of this analysis, AA flagged probesets that targeted truly differentially expressed transcripts (as defined by real time PCR) are simply considered to be False Negatives. Similarly, AA probesets found to represent unchanging transcripts, or probesets targeting transcripts that were undetectable by real time, are reported as True Negatives. The data are summarized in table 1.

**Table 1:** Correct and incorrect predictions for both algorithms

	MAS5 (found/actual number by real time)				RMA (found/actual number by real time)			
	TP	FP	TN	FN	TP	FP	TN	FN

PP	8/8	1/1	0/0	0/0	8/8	1/1	0/0	0/0
PA	8/8	0/0	0/0	0/0	0/0	0/0	0/0	8/8
AARMA	0/0	0/0	4/4	9/9	9/9	4/4	0/0	0/0
AA	0/0	0/0	1/1	4/4	0/0	0/0	1/1	4/4

TP: True Positive. FP: False Positive. TN: True Negative. FN: False Negative. For example, for the PP set, MAS5 and RMA both found 8 TPs out of a total of 8 possible, and made 1 incorrect prediction, resulting in 1 FP.

It can be seen that the both algorithms behave similarly for the PP and AA probesets, and that the key source of discrepancy is for the PA and AA-RMA sets. Over these data, MAS5 made correct predictions for 100% of the PA probesets, and 31% of the AA-RMA ones, while RMA was correct for 0% of the PA probesets, and 69% of the AA-RMA ones.

Four of the 'truly changing' AA flagged probesets were considered to be False Negatives. It is important to appreciate that by flagging these probesets AA, the algorithm is refraining from making a call rather than explicitly calling the probeset as being unchanging (i.e., it is reporting 'don't know' rather than 'no-change'). Thus, although MAS5 fails to make the right call for these data, by reporting 'don't know', it is not getting them wrong either. From this perspective, counting AA probesets as either True- or False-Negatives is a somewhat restrictive interpretation, since doing so fails to recognize the algorithm's ability to avoid making a call when the data is not of sufficient quality to warrant it.

### Latin square data revisited

**Figure 4:**
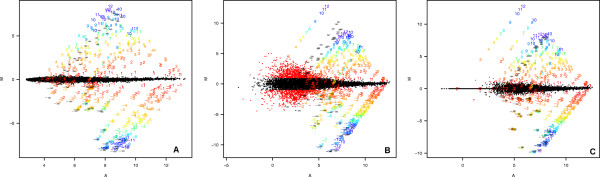
**MA plots for the Latin square data, generated by affycomp [4]**. Points represent probesets targeting transcripts not expected to change, numbers represent probesets targeting transcripts spiked into the dataset at different concentration. The number represents the expected fold change. Fold change is represented by M on the y-axis. A: RMA processed data. B: Raw MAS5 processed data. C: filtered-MAS5 data. Raw MAS5 data suffers from a significant number of False Positives (i.e. probesets recording a differential expression greater than 2-fold), while RMA shows much better performance on these data. Filtering MAS5 data by detection call as described in Methods, significantly reduces the number of False Positives and brings the results much closer to those of RMA.

The high performance of MAS5.0 on these data is perhaps surprising, given that it has previously been reported to perform badly on the Latin square datasets described above [2-4]. Figures 4A and 4B show fold changes for these data, processed using MAS5.0 and RMA, as found by 'affycomp' package in BioConductor [4]. Figure 4C shows the results of filtering the MAS5.0 data by detection call (see also Methods). Much of the low intensity variation is eliminated, significantly reducing the number of False Positives (FPs) and bringing the data closer into line with RMA. This can be quantified using affycomp, a tool designed to score the performance of expression summary algorithms (on the spike-in datasets described above) against a variety of criteria, and to use these scores to provide an unbiased assessment of their relative performance [4]. Metrics are described in detail in [4,15], and are summarized briefly in the methods. Of the 14 metrics reported on the affycomp website, MAS5.0, when filtered as described, marginally out-performs RMA on 6 of them and comes close on a further 3 (table 2).

**Table 2:** Affycomp scores for RMA, GCRMA, MAS5 and MAS5 filtered by detection call

	rma	Gcrma	mas5	filtered mas5
Median SD	0.07	0.06	0.29	0.08
null log-fc IQR	0.13	0.04	0.47	0.01
null log-fc 99.9%	0.40	0.61	4.01	1.25
Signal detect R2	0.90	0.91	0.91	0.88
Signal detect slope	0.68	1.00	0.77	0.77
low.slope	0.20	0.25	0.58	0.25
med.slope	0.71	1.13	0.73	0.79
high.slope	0.80	0.97	0.77	0.77
Obs-intended-fc slope	0.68	1.00	0.77	0.77
Obs-(low)int-fc slope	0.31	0.48	0.64	0.44
low AUC	0.43	0.35	0.00	0.19
med AUC	0.85	0.86	0.00	0.18
high AUC	0.93	0.86	0.00	0.18
weighted avg AUC	0.53	0.48	0.00	0.19
AFP, call if fc > 2	1.71	3.30	2863.36	73.73
ATP, call if fc > 2	32.91	35.40	35.45	34.18
FC = 2, AFP, call if fc > 2	0.24	1.95	2785.10	63.52
FC = 2, ATP, call if fc > 2	10.81	19.57	14.33	12.86

Metrics are described in detail in (Cope et al. 2003) and briefly in the methods. The last four rows record the number of True and False positives. Perfection for the first three rows is 0, and 1 for the next 11 rows. Filtering by detection call significantly improves the performance of MAS5 on the Latin square data.

For these metrics its performance is similar to that of GCRMA. For metrics dependent on accurate reporting of fold change, MAS5.0 outperforms RMA, and is similar in performance to GCRMA. 4 of the 14 metrics (low-, med-, high- and weighted avg AUC) in affycomp are based on ROC curves, generated by considering the number of True Positives (TPs) for a given number of False Positive (FPs). For these, it behaves worse than either RMA or GCRMA. Investigation of individual TP and FP rates reveals that the poor performance of MAS5.0 on the ROC based metrics is due to a very high FP rate. Filtering by detection call, however, does lead to significant improvements: it successfully reduces the number of FPs from 2,863 to 74 (figure 4). When the algorithm is used alongside detection filtering [16], its performance on these Latin square data becomes much closer to that of its peers.

Thus, when filtered by detection call, MAS5.0 performs well, not only on the spike-in datasets described above, but also on real experimental data in which many transcripts are differentially expressed. Further, MAS5.0, when used alongside detection filtering correctly identifies a set of differentially expressed transcripts for which other approaches record low changes. As discussed earlier, these PA probesets are of particular interest because of their potential 'switching' behaviour.

### Biological validation

In total, there are 40 PA probesets with greater than 8 fold differential expression according to MAS5.0, but less than 2-fold by RMA (table 3), and 1,142 when the MAS5.0 threshold is reduced to 2-fold.

**Table 3:** PA flagged probesets with > 8 fold differential expression according to MAS5 and less than 2 fold according to RMA.

Probeset ID	f.c.	ttest	mcf7	mcf10a	gene name
1553494_at	5.03 ± 0.59	0.00	4.05	-0.97	TDH
210833_at	4.69 ± 0.53	0.00	4.37	-0.32	PTGER3
1564699_at	4.56 ± 0.67	0.01	4.10	-0.46	C5orf4
222136_x_at	4.04 ± 0.54	0.00	7.62	3.58	ZNF43
1558687_a_at	3.86 ± 0.67	0.00	4.23	0.38	NA
228811_at	3.84 ± 0.56	0.00	4.33	0.48	PLA2G5
214761_at	3.59 ± 0.56	0.00	4.63	1.04	ZNF423
207886_s_at	3.58 ± 0.25	0.02	4.74	1.17	CALCR
217681_at	3.40 ± 1.28	0.00	5.66	2.26	WNT7B
210100_s_at	3.38 ± 0.98	0.00	6.48	3.11	ABCA2
205501_at	3.34 ± 1.23	0.04	3.58	0.25	NA
243580_at	3.29 ± 0.75	0.01	4.61	1.32	GNA14
214191_at	3.26 ± 0.68	0.00	5.07	1.81	ICA1
243650_at	3.20 ± 1.51	0.03	4.96	1.76	PLEKHH2
231795_at	3.19 ± 1.08	0.00	4.48	1.29	NA
230917_at	3.16 ± 1.19	0.03	4.34	1.18	NA
228090_at	3.13 ± 0.52	0.01	5.86	2.73	NMNAT3
216061_x_at	3.13 ± 0.63	0.00	6.35	3.22	PDGFB
243804_at	3.12 ± 0.57	0.01	4.12	1.00	FLJ32642
204778_x_at	3.09 ± 1.25	0.01	6.09	3.00	HOXB7
241744_x_at	3.08 ± 0.52	0.04	3.63	0.54	NA
226086_at	3.05 ± 0.49	0.00	4.18	1.13	SYT13
1569701_at	3.03 ± 0.47	0.04	3.91	0.89	PER3
225163_at	3.01 ± 0.72	0.01	5.05	2.03	FRMD4A
204885_s_at	-3.22 ± 0.40	0.01	2.70	5.92	MSLN
214587_at	-3.27 ± 0.28	0.03	0.64	3.92	COL8A1
233792_at	-3.31 ± 0.61	0.00	0.51	3.82	NA
205964_at	-3.44 ± 0.45	0.02	1.22	4.66	ZNF426
219983_at	-3.47 ± 0.27	0.00	0.73	4.20	HRASLS
239955_at	-3.47 ± 0.84	0.00	0.48	3.95	NA
1563693_at	-3.50 ± 0.30	0.00	0.13	3.63	NA
1553574_at	-3.59 ± 1.10	0.00	-0.20	3.39	IFNE1
219058_x_at	-3.70 ± 1.22	0.01	2.46	6.16	LCN7
228821_at	-3.75 ± 0.43	0.01	-0.17	3.58	ST6GAL2
1564796_at	-3.87 ± 0.28	0.01	0.80	4.68	EMP1
225372_at	-4.07 ± 0.39	0.00	0.74	4.82	C10orf54
1556773_at	-4.14 ± 1.39	0.00	0.10	4.24	PTHLH
1562648_at	-4.17 ± 0.84	0.00	0.10	4.28	KIAA1212
1558512_at	-4.29 ± 1.38	0.00	-0.32	3.97	NA
1553654_at	-4.45 ± 0.70	0.00	-0.97	3.48	SYT14

f.c.: log2 fold change, ± standard error. t-test: t-test p-score between replicates. MCF7, MCF10a: log2 mean expression level. Probesets marked in bold type were validated by real time PCR.

Space does not permit full consideration of every one; instead we focus on those most highly over-expressed in the MCF7 cells. L-threonine dehydrogenase (TDH) has been identified as a transcribed pseudogene in humans [17]. PTGER3 (EP3) has been shown to be involved in the activation of Src signaling via Prostaglandin E2 (PGE2) [18], and host stromal PGE2-EP3 signaling appears critical for tumor-associated angiogenesis and tumor growth [19]. The MAS5.0 data shows PTGER3 to be upregulated in MCF7 w.r.t. MCF10A. C5orf4 has been identified as a putative tumour suppressor [20], whilst ZNF43 has been shown, in vitro, using Ewing Sarcoma derived EW-1 cells, to be highly expressed in proliferating cells and down regulated in cells induced to differentiate [21]. Treatment of EW-1 cells with antisense oligonucleotides complementary to ZNF43 mRNA was shown to induce morphological differentiation and growth arrest, suggesting a role for ZNF43 in the maintenance of ES cells in an undifferentiated state [21]. The MAS5.0 data shows ZNF43 to be over expressed in MCF7 cells with respect to MCF10A. Over expression of ZNF423 (OAZ) has been shown to lead to elevated Smad6 expression in C2C12 cells [22]. Smad6 is known to be an inhibitor of the BMP signalling pathway and over expression of both OAZ and Smad6 have been shown in pulmonary smooth muscle cells to result in inhibition of BMP4 mediated apoptosis [22]. Both ZNF423 and Smad6 are found to be over expressed in MCF7 w.r.t. MCF10A, and both are flagged as being Present in MCF7, Absent in MCF10A. Calcitonin receptor expression has previously been reported in MCF7 cell lines[23] and Wnt7B has been previously shown to be upregulated in MCF7 [24]. Thus many of PA genes identified by MAS5.0 show behaviour in agreement with previous reports in the literature.

## Discussion

When a simple fold-change threshold is used to define differentially expressed genes, the MAS5.0 expression summary algorithm yields a significant number of false positives, as illustrated in Figure 4B. This is, at least in part, because even if the signal from both sets of replicates is low, the resultant fold change can still be high. However, as shown here, detection calls can be used to successfully filter MAS5.0 data and remove the vast majority of false positives. This is also in keeping with a recent study that found a significant improvement in False Discovery Rate following pre-filtering by detection call [25].

Detection calls also have significant utility when considering transcripts with expression levels close to the limits of detection of the platform. By selecting probesets changing consistently from Present to Absent, it is possible to identify a small but significant set of transcripts that have large fold changes, are positively validated by real time PCR, are consistent with previous reports in the literature, but are not reported as changing by other approaches. Since detection calls aim to identify probesets with poor signal to noise ratio, it is reasonable to expect that the fold-changes calculated for these probesets will be unreliable. The relatively high degree of correspondence, for the PA probesets, between the MAS5 data and the real time results suggests that their signal to noise ratio is still high enough to provide a relatively stable calculation of fold change. The actual magnitude of fold changes calculated using Absent flagged data should, however, be treated with an appropriate degree of caution, and validation by real time PCR is clearly advisable.

In the analyses of both the real time PCR and Latin square datasets described above, probesets flagged AA were treated in the same way as those that were found to be unchanging. A consequence of this is that they were reported as false negatives if subsequent validation found their target transcripts to be differentially expressed. Since the AA flag is better considered as a statement of uncertainty rather than of no-change, this is a stringent interpretation of the data. It is important therefore to note that every real-time validated probesets for which MAS5 was able to report a reliable change was positively confirmed; where discrepancies arose they were restricted to AA probesets for which, by definition, the algorithm was unable to make a reliable call.

Irrespective of how false negatives are defined, no such issues arise in the definition of true- and false-positives. For these, both algorithms performed similarly, each correctly predicting changes for 8/9 of the PP probesets, and both doing well on the PA and AA-RMA set (MAS5 was correct for 100% of the PA probesets, RMA for 69% of the AA-RMA probesets). Note that for both algorithms, the probesets with the largest fold-changes were tested. The low-throughput nature of real time PCR meant that it was not possible to systematically explore the entire AA-RMA and PA subsets of the data, by, for example generating a random sampling of these data and validating these by real time. For this reason, it was not possible to define a threshold below which reported changes were unreliable, and attention must also be paid to the possibility that regression towards the mean might have a certain influence on the correlation coefficients generated for each algorithm [26]. What the study does show, however, is that identifying PA probesets with large fold-changes is a useful strategy for finding differentially expressed transcripts that are not found by other approaches. Overall, these data provide substantial support in favour of the use of MAS5, filtered by detection call, as a rational alternative for identifying differentially expressed transcripts from Affymetrix microarray data.

## Conclusion

The separation of the data into three sets (PP, PA and AA) based on detection call offers an alternative strategy for data analysis that has the potential to reveal additional transcripts, within the PA category, that the other approaches do not find. If a relatively high False Positive rate is acceptable (~30% in this experiment) then it also appears reasonable to include probesets found to have a fold change >2 by RMA and confirmed by MAS5, even if these data are flagged consistently unreliable by detection call.

Both the MAS5.0 expression summary and detection calling algorithms have been criticized, not only for their poor performance on the Latin square data described above, but also for the lack of statistical justification underpinning them. Indeed, the expression summary algorithm relies on a relatively ad hoc treatment of situations where PM probes are of lower intensity than their MM counterparts. It is interesting, therefore, that despite these reservations, both algorithms perform well in practice. This underlines the importance of using real, representative datasets to assess the performance of different algorithms.

Finally, a feature of the MAS5.0 algorithm is its low computational requirements. MAS5.0 considers data on a per-array basis, placing much lower demands on memory than other approaches that must access the entire dataset at once. As microarray datasets are assembled that contain thousands of arrays, computationally frugal approaches such as MAS5.0 become increasingly appealing. These data provide evidence that such advantages may not have to be taken at the expense of biological precision.

## Methods

### Data analysis

All analyses were performed using BioConductor [27]. RMA, GCRMA, MAS5 data were produced using the implementations found in the 'affy' [28], 'gcrma' [8] and 'simpleaffy' [29] BioConductor packages. MAS5 expression calls were generated using the simpleaffy implementation of [1], detection calls with the simpleaffy implementation of [9]. Unless otherwise stated, alpha1 and alpha2 values were 0.05 and 0.065 respectively, the default values for these arrays. All MAS5 data were scaled to a TGT (Target Intensity) of 100. RMA and GCRMA were run using default settings; GCRMA was computed using the full model, making use of MM probes. Kappa coefficients were generated using the 'concord' package in R. Pearson and uncentered Pearson correlation coefficients were calculated in R in the usual way.

### Data sets

#### Cell line comparison data

Expression data were generated by hybridizing RNA taken from MCF7 and MCF10A human cell lines to Affymetrix HGU133Plus2 microarrays. The experiment was performed in triplicate to generate three replicates for each cell line. Full details of the protocols and sample generation can be found in the supplementary data [see additional file 3].

#### Latin square data

RNA was produced by adding varying quantities of 16 known transcripts to a uniform RNA background taken from a common reference pool. The resultant RNA mixtures were used to hybridize a series of microarrays, resulting in a dataset in which the concentration of each of the spikes was known for each of the samples. Full details of the design can be found in [4].

### Real time PCR

Quantitative PCR assays were designed to detect the Affymetrix target sequences for the selected probe sets. Candidates for validation were selected (see figure 1) by eliminating all 'non-standard' probesets (i.e. any that have ids not defined by '* [0–9]_at'). Probesets were selected to represent PP, PA and AA sets, and were chosen across a range of fold-changes. The PA and AA-RMA probesets with the largest fold changes were selected (MAS5 or RMA, respectively). AA and PP probesets were chosen to represent a range of fold changes by defining a set of thresholds (2, 3, 4, 5, 6 fold) and selecting the probesets adjacent to these. Note that a set of controls were also selected to represent genes with 0 fold change – see below. Thus probeset selection was not motivated by any prior knowledge or biological hypothesis. All PP probesets were called Present on all arrays, AA probesets were flagged Absent on at least one sample in each cell line and PA probesets were called Present in all three members of one of the cell lines and Absent in at least one member of the other cell line. Probesets from this group were selected to have large fold changes in MAS5.0 but RMA fold-changes < log2(2). This approach to filtering is similar to that described in [25]. All AA-RMA probesets were flagged AA but had an RMA fold-change > 2. Probesets from this group with the biggest RMA fold change were selected.

qPCR assays were designed using the Exiqon Human Universal Probe Library system. Out of an initial pool of 50 selected transcripts there were 38 for which the Probe Library system was able to design assays. This high fail rate was due to limiting assays to the Affymetrix probe selection regions for each transcript, which are generally around 500 bp long. In some cases the Probe Library software was unable to design an acceptable assay within such a small region. Validation of these 38 assays led to a final list of 35 which were used in the present study.

Labelled probes were purchased from the Exiqon Human Universal Probe Library system [30] (Roche, Switzerland), with amplification primers obtained from MWG (Germany). RNA was extracted from MCF7 and MCF10a cell lines using RNeasy reagents (Qiagen, Germany) and reverse transcribed to cDNA using Taqman Reverse transcription reagents (Applied Biosystems, USA) with random hexamers as primers. Experiments were performed on an ABI 7900 Real Time Sequence Detection System in 384 well format, using an Epmotion 5070 robot (Eppendorf, Germany) for plate set up.

23 of the 78 assays came up after 40 cycles, which we considered to be below the limit of detection. For the purposes of fold-change calculations, the values for these samples were set to 40 cycles. Five potential control genes were selected on the basis of being predicted as unchanging in the array data by both algorithms and the most consistent, Beta 2 Microglobulin (NM_004048), selected for use as a normalisation assay. All reactions were performed in triplicate and the whole experiment was repeated three times. Data analysis was performed using the DDCt method [31].

### P/M/A filtering for AffyComp

The median expression level, I, for all A and M flagged values was calculated for each probeset. Filtering was performed by setting the value for all samples flagged A/M to the median value, I. Thus reliable probesets, consistently flagged P, are left unchanged, whilst unreliable probesets, consistently flagged A, have their values set to a constant for that probeset. Their fold-changes therefore become zero. Probesets changing from A to P will show a fold change calculated between the value for the reliable (P) sample and the median value, I. This approach to filtering is necessary in order to analyze the data using Affycomp (see below), since many of the metrics are sensitive to the number of probesets in the analysis. It is not possible simply to remove probesets from the data. Instead, they must be kept and their values adjusted to prevent their influencing on subsequent calculations. More sophisticated methods of filtering based on the replicate structure of the experiment, are, of course possible, but this was felt to lead to an unfair comparison against other algorithms that make no use of this information.

### AffyComp metrics

The metrics used by AffyComp are fully described in [4,15]. A brief overview is useful here, however. All the metrics are generated by processing the Latin square dataset and comparing the fold-changes and signal intensities produced by the test algorithm to the expected values, given the known concentrations of the target transcripts spiked into each of the arrays. The dataset was sufficiently controlled that the fold-change for every transcript can be predicted between every pair of samples. These data can be used to investigate how good the test algorithm is at reporting zero fold change for transcripts known to be unchanging, for having a linear signal response, for reporting the correct fold change for those that are known to be changing, and for allowing differentially expressed genes to be found by partitioning the dataset on fold change. These are assessed in AffyComp using the following metrics (reported in table 1): **median SD **provides a measure of the standard deviation between replicates in the dataset. A low median SD corresponds to an algorithm that reports data with consistently low variance across the range of expression levels. i.e. perfection is 0. Calculated for unchanging probesets, the **null log fc IQR **and **null log fc 99.9%**, report the interquartile range and 99.9th percentile across the different arrays. For these, unchanging probesets' signal strength shouldn't vary, thus perfection is 0. **signal detect slope & signal detect R2**: the data were such that a plot of observed concentration against expected concentration for the spike probes should have a gradient of 1. The metrics report the slope and the R2 values from a simple linear model fitted to the data. Both should ideally be 1. **low, med, high slope**: fitted slopes, as before, but calculated for probesets targeting low, medium and high concentration targets (the maximum concentration < 4 pM, 4–32 pM and > 32 pM respectively); **obs,-intended-fc-slope and Obs-(low)-int-fc-slope**: fold changes for the spike probesets were calculated between pairs of arrays and compared to the expected fold changes. A plot of these data should result in a graph with a slope of 1. The slope was calculated for all probesets and also restricted to those where the nominal concentration <2 pM. Perfection is 1 for both metrics. **low, med, high, weighted avg AUC**: Expression ratios for each probeset were calculated for every pair of arrays and ordered by their absolute log-ratio. Thus the number of True and False Positives (TPs and FPs) above a given ratio can be found for any pair of arrays, and consequently, the number of TPs for a given FP rate. ROC curves were generated using these data up to a maximum of 100FPs, averaged across all array-pairs and standardized to a maximum height of 1. The ideal ROC curve has an area under the curve (AUC) of 1 and corresponds to the situation where all TPs are found before a single FP is encountered. ROC curves are reported separately for the three concentration groups described above, along with an average weighted by the number of probesets in each group. **AFP, TFP call if fc > 2**: the number of true and false positives found simply by imposing a 2-fold threshold. **FC = 2, AFP, call if fc > 2**: as before, but restricted to probesets where the change is expected to be 2-fold.

## Authors' contributions

SDP Designed real time experiment. EKS, LEE did real time bench work. CLW, CJM did the bioinformatics. CJM, SDP directed the study. CJM wrote the manuscript.

## Supplementary Material

Additional file 1 A comparison of fold changes found by GCRMA and MAS5 for MCF7 and MCF10a cell line data. As figure 1 in the text, but generated using GCRMA instead of RMA. Click here for file

Additional file 2 correlations between microarray and real-time PCR data. Click here for file

Additional file 3 Supplemental protocols. details of microarray protocols. Click here for file
