# Supplementary figures and images for: The utility of MAS5 expression summary and detection call algorithms
Source: BMC Bioinformatics. 2007 Jul 30;8:273. doi: 10.1186/1471-2105-8-273 (PMC1950098; doi:10.1186/1471-2105-8-273)

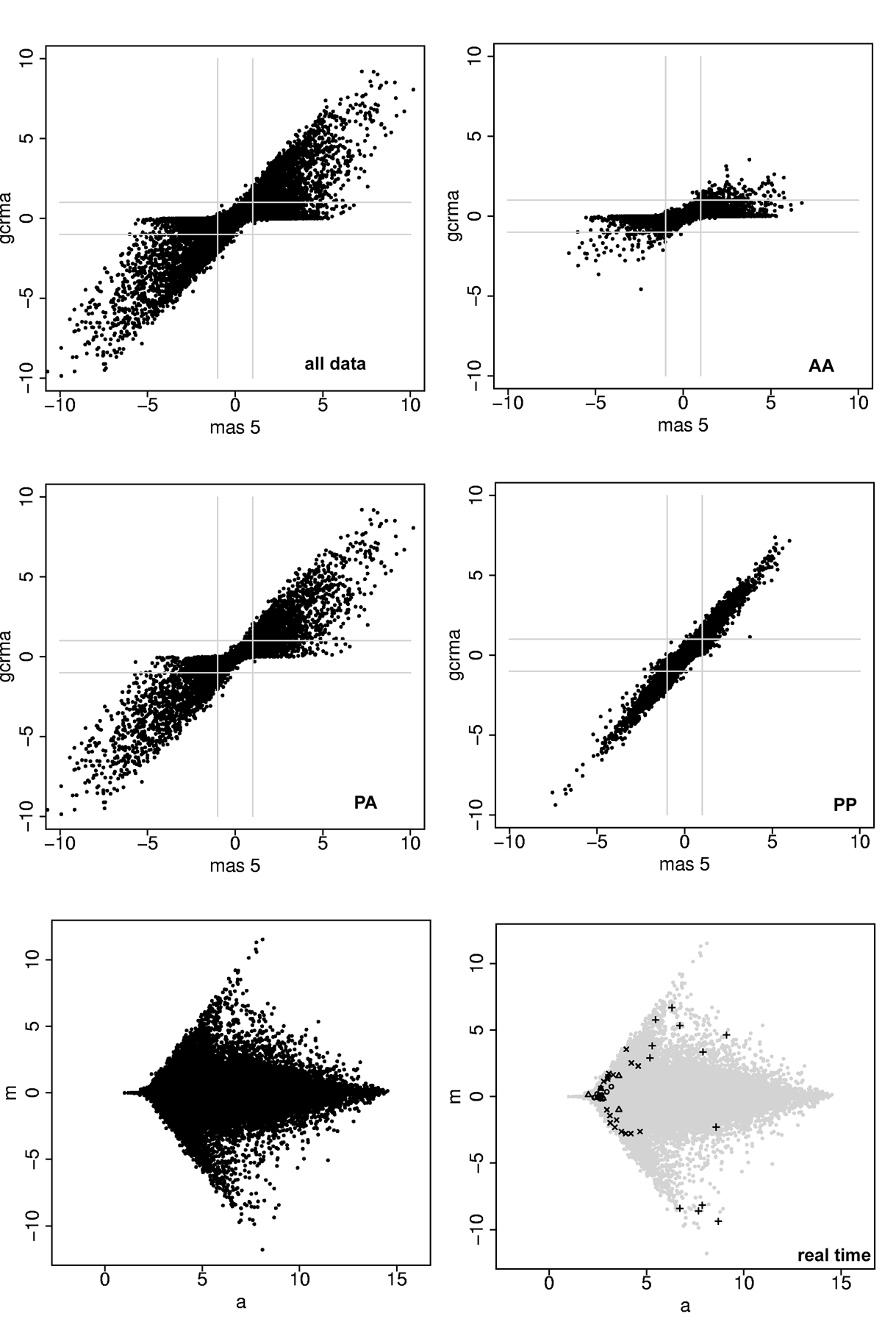

Supplement: Additional file 1 — A comparison of fold changes found by GCRMA and MAS5 for MCF7 and MCF10a cell line data. As figure 1 in the text, but generated using GCRMA instead of RMA. [file 1471-2105-8-273-S1.tiff]
