# Supplementary material for: The utility of MAS5 expression summary and detection call algorithms
Source: BMC Bioinformatics. 2007 Jul 30;8:273. doi: 10.1186/1471-2105-8-273 (PMC1950098; doi:10.1186/1471-2105-8-273)
Supplement: Additional file 2 — correlations between microarray and real-time PCR data. [file 1471-2105-8-273-S2.pdf]

Pearson	PP	AA	PA	AA-RMA
**MAS 5**	0.92	0.72	0.93	0.85
**RMA**	0.93	0.79	0.91	0.86
**CCC**	**PP**	**AA**	**PA**	**AA-RMA**
**MAS 5**	0.92	0.67	0.93	0.67
**RMA**	0.93	0.47	0.32	0.25
