# Supplementary material for: The utility of MAS5 expression summary and detection call algorithms
Source: BMC Bioinformatics. 2007 Jul 30;8:273. doi: 10.1186/1471-2105-8-273 (PMC1950098; doi:10.1186/1471-2105-8-273)
Supplement: Additional file 3 — Supplemental protocols. details of microarray protocols. [file 1471-2105-8-273-S3.pdf]

Cell culture

MCF7 cells were grown in Dulbecco's modified Eagle's medium (DMEM) with 10% (v/v) fetal calf serum (FCS; Invitrogen, Carlsbad, CA, USA), and MCF10A was grown in DMEM/F12 with 5% (v/v) horse serum (Invitrogen, Paisley, UK), 2 ng/mL epidermal growth factor (PeproTech, Rocky Hill, NJ, USA), 0.5 µg/mL hydrocortisone, 0.5 µg/mL cholera toxin, and 5 µg/mL insulin (Sigma, St. Louis, MO, USA). Protein content per cell was similar for the two populations and all experiments were performed on sub-confluent cells cultured at the same density.

Microarray processing

All experiments were performed using Affymetrix HGU133 plus 2.0 oligonucleotide arrays, as described at http://www.affymetrix.com/products/arrays/specific/hgu133plus.affx. Total RNA from each sample was used to prepare biotinylated target RNA, with minor modifications from the manufacturer's recommendations (http://www.affymetrix.com/support/technical/manual/expression_manual.affx). Briefly, 10µg of mRNA was used to generate first-strand cDNA by using a T7-linked oligo(dT) primer. After second-strand synthesis, in vitro transcription was performed with biotinylated UTP and CTP (Enzo Diagnostics, Farmingdale, NY, USA) resulting in approximately 100-fold amplification of RNA. A complete description of procedures is available at http://bioinf.picr.man.ac.uk/mbcf/downloads/GeneChip_Target_Prep_Protocol_CRUK_v_2.pdf. The target cDNA generated from each sample was processed as per manufacturer's recommendation using an Affymetrix GeneChip Instrument System (http://www.affymetrix.com/support/technical/manual/expression_manual.affx). Briefly, spike controls were added to 10µg fragmented cDNA before overnight hybridisation. Arrays were then washed and stained with streptavidin-phycoerythrin, before being scanned on an Affymetrix GeneChip scanner. A complete description of these procedures is available at http://bioinf.picr.man.ac.uk/mbcf/downloads/GeneChip_Hyb_Wash_Scan_Protocol_v_2_web.pdf. Each sample was screened and found to be free from mycoplasma contamination. Additionally, quality and amount of starting RNA was confirmed using an Agilent Bioanalyser. After scanning, array images were manually assessed to confirm scanner alignment and the absence of significant bubbles or scratches on the chip surface. 3'/5' ratios for GAPDH and beta-actin were confirmed to be within acceptable limits (0.99 – 1.01 and 1.01 – 1.05 respectively), and BioB spike controls were found to be present on all arrays, with BioC, BioD and CreX also present in increasing intensity. When scaled to a target intensity of 100 (using Affymetrix MAS 5.0 array analysis software), scaling factors for all arrays were within acceptable limits (1.48 - 2.01), as were background, Q values and mean intensities.
